# Calculated radiation exposure for trauma patients is lower when using the New Injury Severity Score versus the Injury Severity Score to calculate injury severity

**DOI:** 10.1186/cc13233

**Published:** 2014-03-17

**Authors:** M Wagner, J Vonk, C Wichman, A Hegde, J Oliveto

**Affiliations:** 1 Creighton University, Omaha, NE, USA; 2 University of Iowa, Iowa City, IA, USA; 3 UNMC, Omaha, NE, USA

## Introduction

We studied radiation exposure (RE) in trauma patients [1]. To adjust for injury severity the New Injury Severity Score (NISS) or the Injury Severity Score (ISS) can be used to group patients. We sought to determine whether there is a difference in the calculated RE per group when the patients are grouped using either the NISS or ISS.

## Methods

Data collection was as previously described [1]. RE was measured in milliSieverts (mSv). Group analysis was done for both ISS and NISS using the following severity score ranges (SSR) 1 to 8, 9 to 15, 16 to 25, and >25.

## Results

The analysis conducted in previous research [1] was repeated for the data recategorized by NISS. The conclusions were identical; increased NISS is associated with increased radiation exposure. A total of 465 patients fell into different SSR when classified by NISS. The distribution of exposure for each SSR, NISS versus ISS, was compared using Wilcoxon's test. For the range 1 to 8, there was no detectable difference. There was a significant shift in the distribution of exposures for the remaining three ranges (*P *< 0.02). The exposure shift was downward for NISS compared with ISS.

## Conclusion

In this analysis, with the exception of patients in groups 1 to 8 there was a significant decrease in RE in the NISS groups when compared with the compared ISS group. Further analysis is needed to determine the cause for this change and whether this difference will be clinically significant.
